# Anchoring Secreted Proteins in Endoplasmic Reticulum by Plant Oleosin: The Example of Vitamin B12 Cellular Sequestration by Transcobalamin

**DOI:** 10.1371/journal.pone.0006325

**Published:** 2009-07-22

**Authors:** Laurent Pons, Shyue-Fang Battaglia-Hsu, Carlos Enrique Orozco-Barrios, Sandrine Ortiou, Celine Chery, Jean-Marc Alberto, Martha Ligia Arango-Rodriguez, Dominique Dumas, Daniel Martinez-Fong, Jean-Noel Freund, Jean-Louis Gueant

**Affiliations:** 1 Inserm U954, Faculté de Médecine, Nancy-Université, Vandoeuvre-les-Nancy, France; 2 Department of Pediatrics, Duke University Medical Center, Durham, North Carolina, United States of America; 3 Department of Physiology, Biophysics and Neuroscience, CINVESTAV, Mexico D.F., Mexico; 4 Plateforme IBISA d'imagerie et biologie cellulaire (PTIBC), UMR CNRS 7561, FR CNRS/Inserm 3209, Faculté de Médecine, Nancy-Université, Vandoeuvre-les-Nancy, France; 5 INSERM, U682, Strasbourg, France; AgroParisTech, France

## Abstract

**Background:**

Oleosin is a plant protein localized to lipid droplets and endoplasmic reticulum of plant cells. Our idea was to use it to target functional secretory proteins of interest to the cytosolic side of the endoplasmic reticulum of mammalian cells, through expressing oleosin-containing chimeras. We have designed this approach to create cellular models deficient in vitamin B12 (cobalamin) because of the known problematics associated to the obtainment of effective vitamin B12 deficient cell models. This was achieved by the overexpression of transcobalamin inside cells through anchoring to oleosin.

**Methodology:**

chimera gene constructs including transcobalamin-oleosin (TC-O), green fluorescent protein-transcobalamin-oleosin (GFP-TC-O) and oleosin-transcobalamin (O-TC) were inserted into pAcSG2 and pCDNA3 vectors for expression in sf9 insect cells, Caco2 (colon carcinoma), NIE-115 (mouse neuroblastoma), HEK (human embryonic kidney), COS-7 (Green Monkey SV40-transfected kidney fibroblasts) and CHO (Chinese hamster ovary cells). The subcellular localization, the changes in vitamin B12 binding activity and the metabolic consequences were investigated in both Caco2 and NIE-115 cells.

**Principal findings:**

vitamin B12 binding was dramatically higher in TC-O than that in O-TC and wild type (WT). The expression of GFP-TC-O was observed in all cell lines and found to be co-localized with an ER-targeted red fluorescent protein and calreticulin of the endoplasmic reticulum in Caco2 and COS-7 cells. The overexpression of TC-O led to B12 deficiency, evidenced by impaired conversion of cyano-cobalamin to ado-cobalamin and methyl-cobalamin, decreased methionine synthase activity and reduced S-adenosyl methionine to S-adenosyl homocysteine ratio, as well as increases in homocysteine and methylmalonic acid concentration.

**Conclusions/Significance:**

the heterologous expression of TC-O in mammalian cells can be used as an effective strategy for investigating the cellular consequences of vitamin B12 deficiency. More generally, expression of oleosin-anchored proteins could be an interesting tool in cell engineering for studying proteins of pharmacological interest.

## Introduction

**Figure 1:**
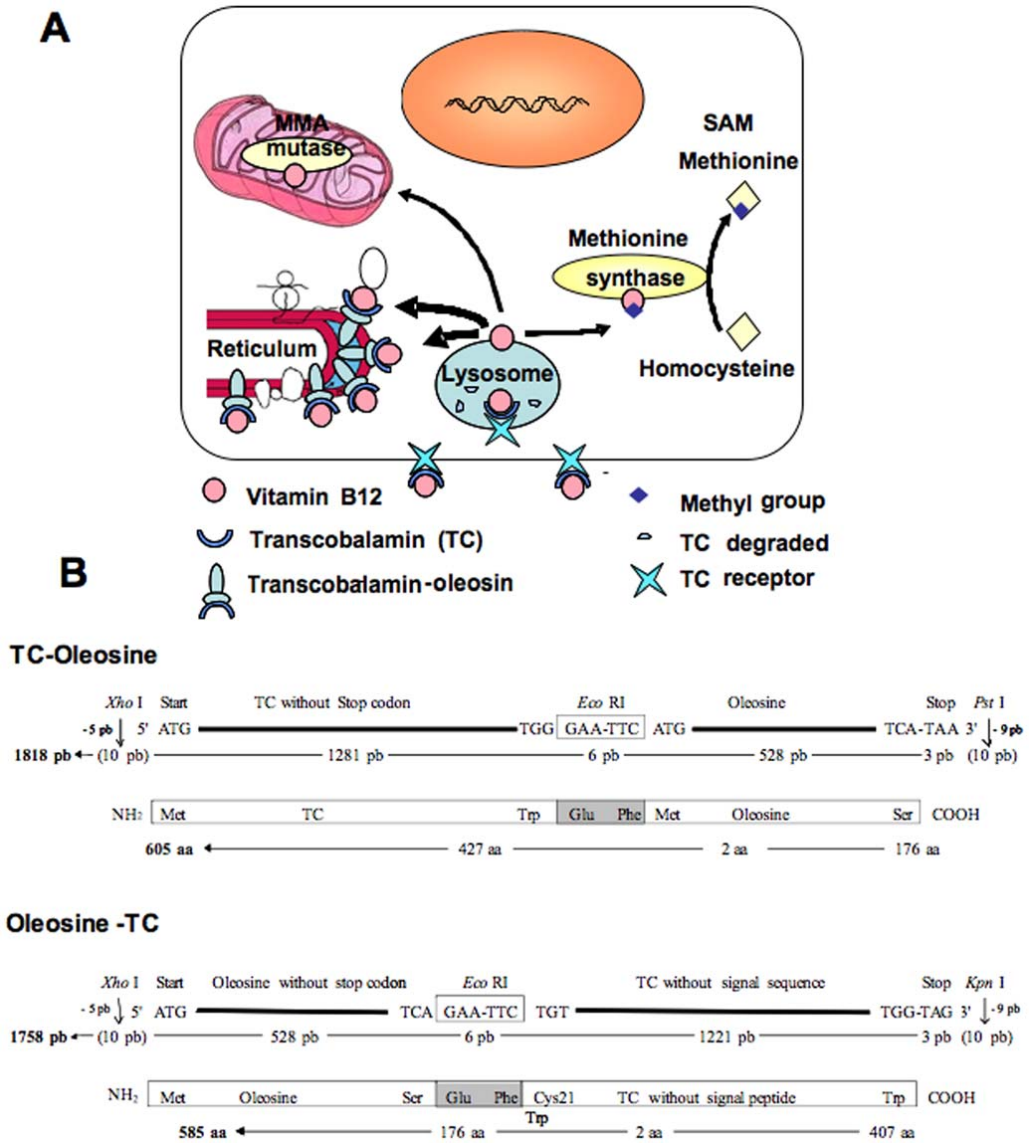
**(A)** Experimental model of vitamin B12 intracellular sequestration by the transcobalamin-oleosin chimeric protein. **(B)** The schematics of the transcobalamin-oleosin (TC-oleosin) and oleosin-transcobalamin (oleosin-TC) cDNA constructs in the plasmid pAcSG2.

Targeting functional secretory proteins to cell membranes may be a useful tool for research in cell biology, genetics, and disease treatment. Previously, various strategies have been used to redirect or anchor either secretory or intracellular proteins to cell surface to uncover novel protein function and properties [1], [2]. An alternate strategy is to localize/limit functional secretory proteins to the intracellular membrane in order to study the consequences of this and eventually reveal the associated molecular mechanisms. Oleosin is a plant oil body protein targeted by its central hydrophobic to the endoplasmic reticulum (ER) [3], [4]. Evidence has indicated that when expressed in plant tissues lacking oil body, oleosin accumulates in ER membrane [3], [5]. The localization pattern is conserved even when it is heterologously expressed in eukaryotic cells, including those of mammalian origin [6]. Our idea was therefore that oleosin could be a tool for targeting secreted proteins to the cytoplasmic side of the ER through the making of oleosin-fused proteins. We first tested this method in cultured mammalian cells using transcobalamin (TC), a secretory protein, and the green fluorescent protein (GFP)-fused TC-oleosin (figure 1A). TC is the secreted carrier protein of vitamin B12 that binds to vitamin B12 with the highest specifity and affinity [7], [8]. Anchoring TC to ER was expected to sequester vitamin B12 within the cells (figure 1A). The minute amount of vitamin B12 needed by cells can be provided by the receptor endocytosis of TC-bound B12 from blood (TC) [7], [8]. The TC-bound B12 that can be provided to cells by fetal calf serum is in order of 5 fmol/ml and the apical release of endogenous TC in order of 2 fmol/cm2/h in confluent caco-2 cells cultivated in standard conditions [8]. Intracellular, B12 is converted into methyl-cobalamin and adenosyl-cobalamin, the cofactors of the cytoplasmic methionine synthase (EC 2.1.1.13) and the mitochondrial enzyme L-methylmalonyl-coenzyme A mutase (EC 5.4.99.2), respectively (figure 1A).

We are interested in studying the effect of B12 deficiency in an effective cultured cell models [10], [11], since it is known that only a minute amount of vitamin B12 is needed by animal cells and in normal culture systems a sufficient quantity of it is provided by either the autocrine synthesis of the TC or by the fetal calf serum added to the culture medium for the maintainess of the cellular growth [8], [12]. This is why in normal culture system vitamin B12-dependent metabolic pathways continue to function even in the absence of exogenously added vitamin B12 [8], [12]. Up to now, no efficient vitamin B12 deficient cell model exists for mechanistic studies, except the use of anti-TC antibodies for impairing the growth of leukemia cells [12]. In humans, two crucial metabolic pathways require vitamin B12 as cofactors [13]. One rearranges MMA to succinyl CoA, and is catalyzed by methylmalonyl CoA mutase in mitochondria. The other produces methionine, the precursor of S-adenosyl methionine (SAM), by remethylation of homocysteine (Hcy) and is catalyzed by methionine synthase in the cytoplasm. SAM is the universal methyl donor of the transmethylation reactions involved in methylation of DNA, lipids and proteins. Vitamin B12 deficiency leads to the impairment of these pathways, which results in accumulation of MMA and Hcy, megaloblastic anemia and central nervous system abnormalities. It is not clear if vitamin B12 exerts its function only through its role as the co-factor of two enzymes mentioned above. For example, it influences the status of pro-inflammatory cytokines in central nervous system and modulates the translation of methionine synthase by the internal ribosome entry site (IRES) [14], [15]. These examples underscore the need to establish effective cell models for the studying the molecular mechanisms of vitamin B12. We present below several lines of evidence that validate the localization of TC-Oleosin chimera in endoplasmic reticulum of the cells in producing intracellular sequestration of vitamin B12.

## Results

**Figure 2:**
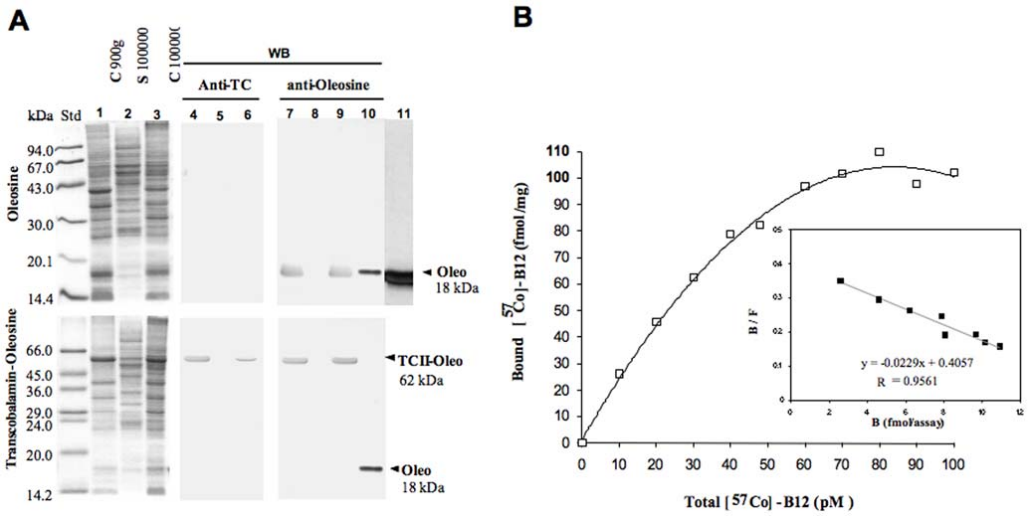
**(A)** Characterization of the recombinant oleosin (top) and transcobalamin-oleosin (bottom) chimeric proteins; SDS-PAGE (12.5%) (lanes 1–3, 10) and Western blot (WB) (lanes 4–9) of subcellular fractions (pellets and supernatant from differential centrifugation) from *Sf*9 cells infected with recombinant baculovirus expressing either peanut oleosin or the transcobalamin-oleosin chimeric protein. Lanes 1, 4, 7: 900 g pellet, lanes 2, 5, 8: 100 000 g supernatant, lanes 3, 6, 9: 100 000 g pellet, lane 10: 85 ng purified peanut oleosin and lane 11: autoradiography of [^14^C]-leucine-labeled oleosin expressed in rabbit reticulocyte lysate. **(B)** Saturation curve of vitamin B12 binding of transcobalamin-oleosin (TC-O) in membrane fraction of lysed sf9 insect cells and corresponding Scatchard plot (inlet).

**Figure 3:**
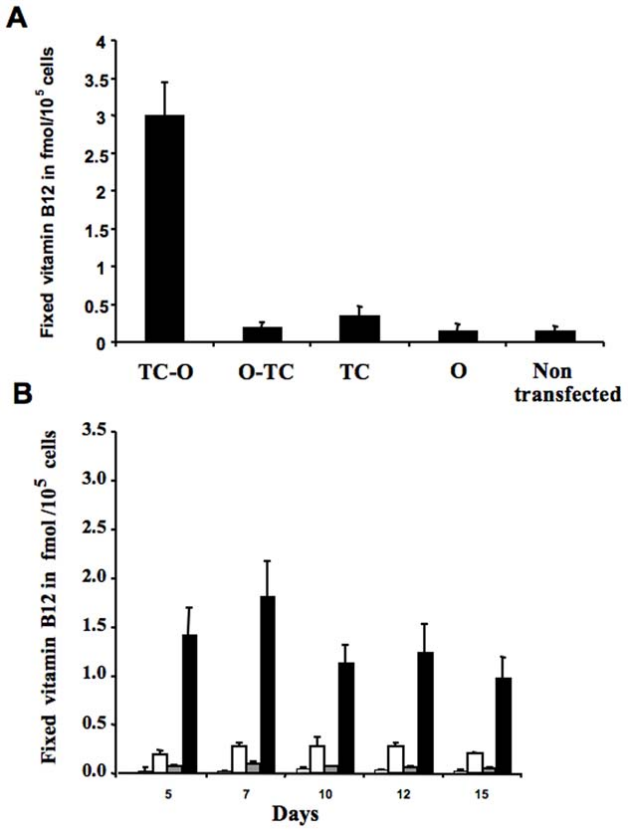
**(A)** Vitamin B12 binding capacity (cyano[^57^Co]Cbl) of membrane fraction (100 µg proteins per assay) from Caco2 cells, 72 hrs after transient transfection with various recombinant pcDNA3 plasmids expressing transcobalamin (TC), oleosin (O) and the chimeric proteins transcobalamin-oleosin (TC-O) and oleosin-transcobalamin (O-TC). **(B)** Vitamin B12 ([57Co]-labeled) binding capacity of Caco2 cells of the intact and lyzed (Caco2 cells were lysed prior to the addition of vitamin B12 or used intact), at days 5–15 of culture, after stable transfection with a recombinant pcDNA3 plasmid expressing the chimeric protein transcobalamin-oleosin. Exponential and stationary phases were evaluated at days 5–7 and days 10–15, respectively. Bars from left to right for each time: non-transfected intact cells, non-transfected lysed cells, TC-O transfected intact cells, TC-O transfected lysed cells.

**Figure 4:**
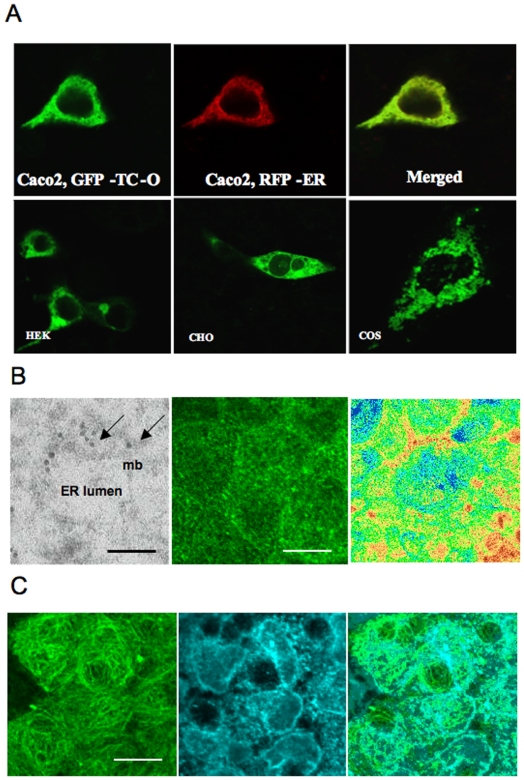
**(A)** Confocal microscopic examination of the localization of the green fluorescent protein-transcobalamin-oleosin (GFP-TC-O) chimeric protein 24 hrs after transient transfection of Caco2, human embryonic kidney cells (HEK293), Chinese hamster ovary (CHO) and COS-7 cells. In Caco2 cells, GFP-TC-O co-localization was detected with an ER-targeted red fluorescent protein (pDsRED2-ER, Clontech USA) (merged). **(B)** Left: Transmission electron microscope examination of the chimera detected immunologically by a Gold conjugate (arrows) (left). Calibration bar = 0.1 µm. Abbreviations: mb, membrane, ER, endoplasmic reticulum. Middle: confocal image of immunolabeling of megalin in apical surface of O-TC caco-2 cells. Right: relative fluorescence intensity (low, red; high: blue) of megalin on apical area. Calibration bar = 18 µm. **(C)** Left and middle: Confocal image of immunolabeling of megalin (Alexa488 coded in green, left channel) and calnexine (Alexa555 coded in cyan, right channel). Right: Merged. Calibration bar = 18 µm.

**Figure 5:**
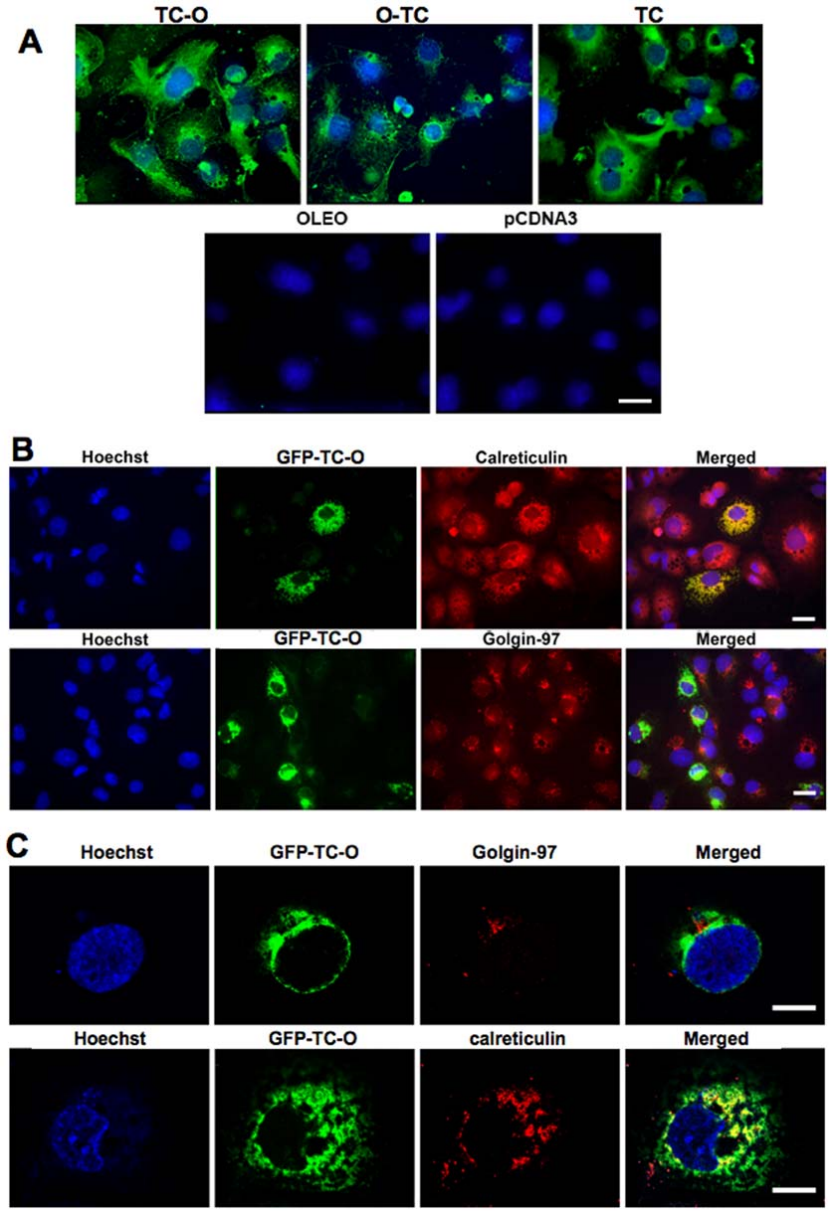
**(A)** Confocal microscopic examination of transcobalamin in COS-7 cells transiently transfected with lipofectamine. The cells were transfected with one of the following pCDNA3-plasmids: TC-O coding for transcobalamin-oleosin (TC-O), O-TC coding for oleosin-transcobalamin (O-TC), TC coding for transcobalamin (TC), O coding for oleosin (O), and the empty plasmid (pCDNA3). The immuno-fluorescence was done with a goat polyclonal antibody to TC and a donkey antigoat IgG fluorescin labeled. Cell nuclei were counterstained with Hoechst 33258. Calibration bars = 20 µm. **(B and C)** Co-localization of the protein GFP-TC-O with endoplasmic reticulum in transient transfected Cos-7 cells using lipofectamine. The cells were transfected with the plasmid GFP-TC-O coding for GFP-transcobalamin-oleosin (GFP-TC-O). The immuno-fluorescence was done with a mouse monoclonal antibody to the human golgin-97 or a rabbit polyclonal antibody to calreticulin. The secondary antibodies were a donkey IgG anti-mouse TRITC labeled or a donkey IgG anti-rabbit TRITC labeled. Cell nuclei were counterstained with Hoechst 33258. Calibration bars = 20 µm.

The peanut oleosin sequence was determined previously [9]. This oleosin was used as a template for constructing the oleosin related plasmids. We ascertained first that oleosin gene could be expressed properly in animal cells by subcloning the peanut gene into pSP64 polyA plasmid for in vitro expression in rabbit reticulocyte lysate. The expressed ^14^C oleosin was immunoadsorbed by rabbit anti-oleosin antibody on Protein A-Sepharose®. After SDS-PAGE, the autoradiograph showed a ^14^C-labeled band at the same 17 kDa molecular size as the native protein (Figure 2B). We then evaluated the expression of the chimeras using pAcSG2 and pcDNA3 plasmids in sf9 insect cells and in mammalian cell lines, respectively. The cells were transiently transfected with each of the recombinant plasmids to quantify the vitamin B12 binding in both intact and lyzed cells. The characterization of vitamin B12 binding of TC-O chimera in membrane fraction of the lyzed sf9 cells showed a Ka of ∼0.02 pmol/L (Figure 2B). Comparing the lyzed and intact cells expressing TC-O chimera, we unrevealed a twelve-fold difference in the B12 binding capacity of the Caco2 cells, with the lyzed cells displaying the much higher binding capacity (Figure 3A). We also found a dramatic difference in cobalamin binding between the O-TC and the TC-O chimeras in intact and lysed Caco2 cells (Figure 3A,B). The same difference was found in sf9 and NIE-115 cells (data not shown). The binding B12 capacity in the membrane fraction was similar in O-TC transfected cells and WT cells treated by sonication, suggesting it was not influenced by the disruption of the ER lumen (1200±49 *vs*. 1180±68 cpm/µg protein). The vitamin B12 binding ability of transfected cells remained stable during continuing culture condition for 15 days (Figure 3B). The binding difference between intact and lyzed TC-O cells was similarly evidenced at days 5–7 and days 10–15, indicating that most of the expressed protein resided within intracellular membranous structures in exponential and stationary growth phases. With confocal microscopy, we subsequently examined the expression of TC-O in Caco2, NIE-115, HEK, COS-7 and CHO cells using GFP-TC-O (figure 4A). The co-localization of the GFP-TC-O with the ER-target red fluorescent protein suggested that TC-O expression is limited to the reticulum. The localization of the chimera in the membranes of endoplasmic reticulum was confirmed by its immunological detection with a Gold conjugate in microscopy electron microscope immunocytochemistry (figure 4B). The expression of TC in ER did not modify the cell targeting of multiligand proteins that share affinity for TC. In particular, the localization of megalin was maintained in the apical surface of caco-2 cells, in confocal microscopy. In addition, we did not observe any co-localization of megalin and calnexin, in confocal microscopy of Caco2 cells expressing the chimera (figure 4B,C). Further confocal microscopic examination with anti-transcobalamin immunofluorescence of the COS-7 transiently transfected with the pCDNA constructs confirmed the results; in cells transfected with pCDNA-TC-O and pCDNA-O-TC, TC was mainly localized in intracellular membranes; in contrast, tranfection with pCDNA3-TC produced a diffused expression of TC in the whole cytoplasm while no TC was detected in cells transfected with pCDNA3-oleosin and empty pCDNA3 (figure 5A). More confocal analysis revealed the co-localization of GFP-TC-O with calreticulin, a protein of ER membrane [16] but not with golgin 97, a protein of Golgi apparatus [17]; these results asserted the ER localization of the oleosin-anchored proteins.

**Figure 6:**
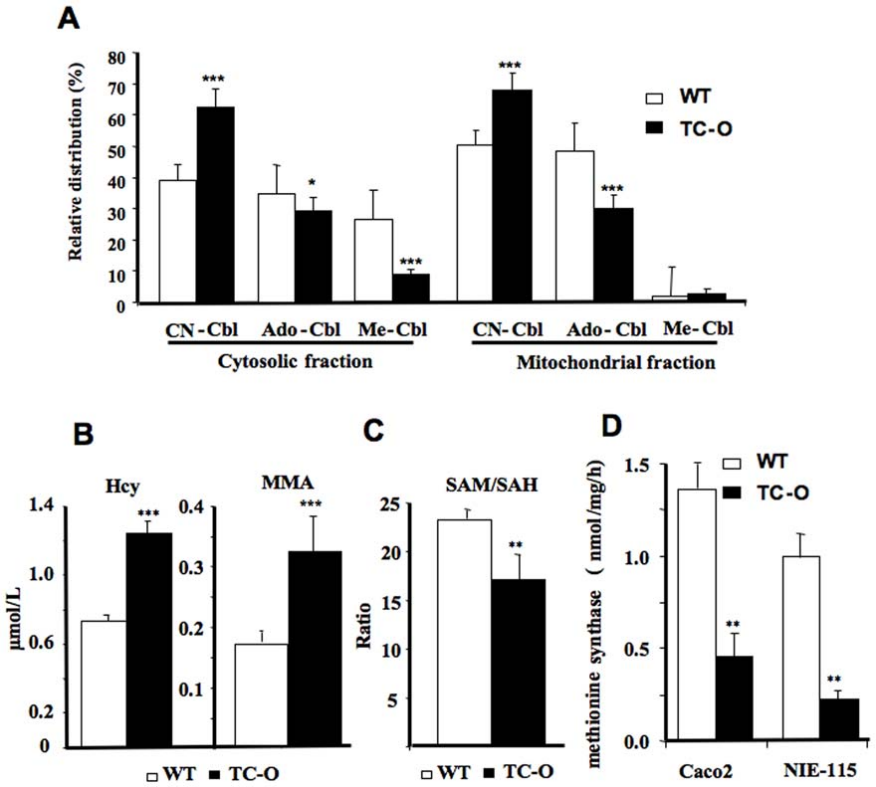
**(A) Intracellular conversion of exogenous [^57^Co]-labeled B12 (cyano-cobalamin, CN-Cbl)** into methyl-cobalamin (Me-Cbl) and ado-cobalamin (Ado-Cbl) in the cytosolic and mitochondria enriched fractions. **(B)** Homocysteine (Hcy) and methylmalonic acid (MMA) concentrations. **(C)** S-adenosylmethionine/S-Adenosylhomocysteine ratio (SAM/SAH) in TO and wild type NIE-115 cells. **(D)** Activity of methionine synthase in TO transfected and wild type NIE-115 and Caco2 cells in exponential growth. Mean±S.D. of sextuplet assays are given. ****: p<0.0001, **: p<0.001, *: p<0.001.

We then examined the consequences of the B12 intracellular sequestration in stably transfected Caco2 and NIE-115 cells. By adding radioactive [^57^Co]-labeled B12 (cyano-cobalamin) to the culture medium, a dramatic decrease in its intracellular conversion to methyl-cobalamin and ado-cobalamin was uncovered both in the cytosolic and the mitochondria enriched fractions of the TC-O expressing cells (Figure 6A). LC-MS/MS analysis of the cellular homocysteine content and the secreted methylmalonic acid associated with TC-O cell culture showed significant changes, compared to the WT cells (Figure 6B, p<0.0001 and p<0.0001, respectively); a reduction in the S-adenosylmethionine (SAM)/S-Adenosylhomocysteine (SAH) ratio was also observed (p = 0.0023, compared with wild type cells) (Figure 6C). We scrutinized the activity of the B12-dependent cytosolic enzyme methionine synthase and found a largely decreased methionine synthase activity in both TC-O transfected Caco-2 and NIE-115 cells (Figure 6D). These results indicate, at least in the cell lines tested, that the cytosolic bioactive B12 (methyl-cobalamin) and the B12 dependent synthesis of methionine, key amino acid needed for cell growth, are diminished in TC-O cells.

## Discussion

Oleosin is a protein originated from plant that has been used as a carrier to produce protein of biological interest in plants [18], [19]. Our approach here in using oleosin as an intracellular anchor for secretory protein in mammalian cells is oriented from another perspective, focusing more on the targeting ability of the protein oleosin. The technology is meant to work in the mammalian system [6]. Because of its unique structure, Oleosin serves as an interfacial molecule to stabilize the oil droplets within the hydrophilic cytosol. In Plants, it is targeted to oil bodies via the endoplasmic reticulum and consists of a lipid-submerged hydrophobic domain H that is flanked by two cytosolic hydrophilic domains N and C [3], [4], [5]. Molecular modeling of oleosins has suggested a configuration with exposing N- and C-terminal domains in cytosol and a central hairpin conformation that anchors the molecule within the lipophilic core [3], [4], [5], [20]. This configuration allows for the positively charged residues within the flanking domains of the oleosin to interact with the negatively charged phosphate groups of the oil body phospholipid monolayer [20]. The interaction between the oleosin and the phospholipid monolayer causes the surface of the oil body to become enveloped in a protein shell [20], [21]. This unique amphipathic structure of the oleosin molecule appeared to be maintained in mammalian cells [6]. This was confirmed in our model, where the fusion protein worked as a vitamin B12 chelator in the cytosolic face of reticulum membrane, as illustrated by the B12 binding experiment in lyzed cells (Figure 3).

The chimeric GFP oleosin TC protein was colocalized with the ER-targeted red fluorescent protein and the ER-bound calreticulin (Figure 4, 5). These results indicated that adding another protein to the N-terminal end of the oleosin protein (through the making a fusion protein) did not alter its intracellular localization. These results confirm previous structural-function studies which suggest the central hydrophobic domain to be necessary for ER targeting, while the N- or C-terminal domain being of no consequence to its membrane localization [6], [20], [21]. Oleosin locates also in oil bodies when ectopically expressed both in yeast and mammalian cells, which are able to produce them [4], [5], [6].

The rationale behind expressing this oleosin fusion protein was to immobilize TC within cells. TC, under normal physiological condition, is synthesized by various cells including polarized epithelial cells such as Caco2 cell and is secreted into plasma in a constitutive manner [7]. Functionally, the plasmatic TC is to bind the circulating vitamin B12. The bound TC then is internalized via receptor-mediated endocytosis [7]. Depending on the cell types, the TC could either be degraded and the released vitamin B12 utilized, or exported out untouched via transcytosis [8]. We expected that the over expression of this immobilized vitamin B12 binding protein would serve as an intracellular cage for vitamin B12. Because the requirement of vitamin B12 for growth and differentiation, although essential, is extremely low, with uncontrollable sources of vitamin B12 in cell culture, one cannot easily evaluate under classical experimental condition how a change in the vitamin B12 level affects cell functions [12]. For example, 10% fetal calf serum provides about 5 fmol/ml of TC bound B12 in our cell culture conditions of caco-2 cells [12]. The endogenous synthesis of TC may also help to internalize the vitamin B12 provided by the culture medium [12]. With the expression of TC-O, we were able to observe the metabolic consequences of vitamin B12 intracellular sequestration, namely a decrease in the intracellular conversion of B12 and the subsequent reduced methionine synthase activity which resulted in increases of Hcy and MMA, and a decrease SAM/SAH ratio. The inclusion of oleosin appeared necessary since evidently, the expression of TC itself offered no significant increase in the amount of vitamin B12 sequestered within cells (Figure 3). We also demonstrated the importance of the position in which oleosin was integrated in the chimeras. TC-O was a very efficient chelator of cobalamin while O-TC was no different than the either wild type non-transfected cells or cells expressing TC alone. This could indicate that integration of TC in the N-terminal of the oleosin impaired the B12 binding capacity of the chimer protein, probably by modifying the conformation of the C-terminal domain of TC [22]. Indeed, TC has a two-domain architecture, with an N-terminal barrel and a smaller C-terminal domain, vitamin B12 being buried inside the domain interface [22]. Another hypothesis could be that the TC part of the O-TC chimera was expressed in the lumen of reticulum and the TC part of the TC-O chimera in the cytosolic face. However, this hypothesis disagrees with the topology of oleosin in reticulum membrane, since oleosin adopts a unique conformation in which the H domain resides completely within the lipid bilayer and is flanked by the hydrophilic N and C domains facing the cytosol [23], [24], [25]. Mutagenesis experiments showed that reticulum membrane topology of oleosin is constrained by its long hydrophobic H domain rather than by the N and C domains [23]. In addition, deletion of the N or C domains indicated that the H domain is the only critical component for reticulum targeting [25]. Our results are consistent with these data. The B12 binding capacity of the membrane fraction was similar in O-TC transfected cells and WT cells after sonication of cell lysate, a treatment that produces a disruption of reticulum lumen. This suggested that the lack of intracellular B12 binding capacity in O-TC tranfected cells was not related with a orientation of the TC part of the chimera in the ER lumen. Finally, our plasmid constructs included the TC sequence without its N-terminal signal peptide at the 3′ end of the oleosin sequence, making the translocation of the C domain-TC part of the chimera in the reticulum lumen unlikely [24].

Since TC is a ligand of megalin and megalin is an actor of B12 uptake in epithelial cells [26], a possible effect of the chimera in caco-2 cells could be to retain megalin in the endoplamic reticulum. The subsequent effects would be to influence its apical expression and B12 uptake. Our results in confocal analysis of megalin showed that its localization was maintained in the apical surface of caco-2 cells and that it was not retained in the endoplasmic reticulum (figure 4B,C), making unlikely the presence of an interaction with the chimera.

Our model was adapted to examine the complex consequences of B12 impaired metabolism in cells, in particular to investigate whether cobalamin has other effects than through the two known B12-dependent reactions (methionine synthase MS and MMA mutase). Alternative strategies for producing cell deficiency in vitamin B12 would be more focused on one of the many actors involved in the internalization, intracellular transport and metabolism of the vitamin. Among these strategies are the uses of anti-TC antidoby [12] and of siRNA. The latter should be targeted and equally efficient on the different actors involved in endocytosis of vitamin B12. In the case of digestive epithelial cells, these actors are cubilin, amnionless, megalin, TC receptor and asialoglycoprotein receptor [26], [27], [28]. In addition, the “knocking down” approaches produce pitfalls related to efficiency, stability, specificity, side effects [29].

In conclusion, oleosin has already been used as a tool of molecular farming for the production of pharmaceutical proteins in plants. Our approach was to use it as an anchor for secretory proteins in cell culture and in vivo, as illustrated by the functional expression of TC-oleosin. The heterologous expression of TC-O in mammalian cells can be an effective strategy for investigating the cellular consequences of vitamin B12 deficiency, in particular in relation with epigenetics, neurodegenerative mechanisms and carcinogenesis, using the anti-TC at a dilution of 1∶60 dilution. More generally, this strategy may be used with other functional proteins.

## Methods

### Reagents

Methionine, homocysteine, homocystine, homocysteine-thiolactone, L-cystine, S-adenosyl-methionine, hydroxocobalamin, protease inhibitor cocktail and Neutral Red were obtained from Sigma (St Louis, MO, USA). Cyanocobalamin was provided by Aguettant (Lyon, France). [^14^C]Methyl tetrahydrofolate was purchased from Amersham Biosciences (Orsay, France) with a specific activity of 57 µCi/µmol. Dulbecco's Modified Eagle's Medium and Dulbecco's Modified Eagle's Medium without L-methionine were from Gibco Invitrogen Corporation (Cergy Pontoise, France). All other chemicals were of cell culture grade.

### Oleosin cDNA preparation

cDNA of transcobalamin gene (TC) was prepared as described.^8^ The peanut oleosin gene (O) sequence was previously determined by us [9]. The oligo(dT)_15_-primed cDNA produced by AMV reverse transcription (Boerhinger Mannheim, Germany) of the total RNA from mature seeds was used for PCR amplification of the complete peanut oleosin open reading frame. Primers for PCR were designed to include a PstI site and a vertebrate Kozak consensus sequence at the 5′ end and a XbaI site at the 3′ end of the coding sequence.

### Expression in rabbit reticulocyte lysate

The peanut gene 560-bp PCR product with the Kozak consensus sequence was subcloned into the EcoRI/PstI restriction sites of pAcSG2 vector for in vitro expression in rabbit reticulocyte lysate (TNT® SP6 Coupled Reticulocyte Lysate System, Promega). The expressed ^14^C oleosin was immunoadsorbed by rabbit anti-oleosin antibody on Protein A-Sepharose® CL-4B (Pharmacia, Saint-Quentin en Yvelines, France).

### Expression in the Sf9-baculovirus system

The recombinant baculovirus (1 µg) was used for translation of recombinant pAcSG2 plasmids by an Sf9-baculovirus expression system. The cells were infected with the recombinant virus in 75 cm^2^ Petri plates (15.10^6^ cells per plate, 3.5 ml of virus dilution in culture medium with MOI at 10). They were collected 72 hours after infection, sonicated 2 times for 20 s using a cell disruptor (B-30, Branson Sonic Power Co., USA) and subjected to fractionation at 4°C by a two-step centrifugation at 900 g for 3 min and at 100,000 g for 1 hr, respectively. The 100,000 g pellet and supernatant corresponded to the membrane-enriched fraction and to the cytosol fraction respectively.

### In vitro expression in mammalian cells

The cDNA from the oleosin gene and the transcobalamin gene were ligated into the BamHI/XbaI site of a pcDNA3 vector (Invitrogen, Cergy Pontoise, France) to generate pTC-O. The resulting plasmid contained the cytomegalovirus (CMV) promoter, the Kozac sequence, a polylinker cloning site containing the transcobalamin-oleosin (TC-O) sequence, and a SV40 polyadenylation signal. This expression cassette was flanked by resistance genes directed towards ampicillin and neomycin. For pCDNA_3_-oleosin, a PCR product, which included a 5′ EcoRI site and a 3′ XbaI site around the oleosin sequence, was inserted into the corresponding MCS sites of the vector. For TC-O, the PCR product containing the complete human TC coding region without its N-terminal signal peptide and its stop codon was inserted at the 5′ end of the oleosin sequence between the BamH1 and EcoRI sites of the vector. For O-TC, the oleosin coding sequence was first inserted without its stop codon in pCDNA_3_ between the BamH1 and EcoRI sites; the TC sequence without its N-terminal signal peptide was then inserted at the 3′ end of the oleosin sequence at the EcoRI/XbaI sites. For green fluorescent protein-TC-O (GFP-TC-O), the PCR product containing BamHI sites at both the 5′ and 3′of the GFP without its terminal stop codon was inserted into the pCDNA3-TC-O. The plasmid was selected based on the expression of green fluorescence upon transfection in cells. The cell lines were transfected with the pTC-O and pO-TC plasmids by the Exgen method (Euromedex, France). Two micrograms of plasmid DNA solution was added to the Exgen solution (100 pmol in 150 mM NaCl solution) by slow dropwise addition with continuous mixing. The resulting complexes were incubated at room temperature for 10 min before incubation with cells. 48 h after transfection, cells were sub-cultured and selected with 1.1 mg/ml of G418. After 3 weeks of selection, the clones were isolated and amplified in the presence of 1.1 mg/ml G418. To confirm the expression of TC-O and O-TC fusion proteins by cells, cellular RNA was isolated from the cells. Cells were rinsed with PBS and directly extracted with the RLT buffer (300 L/well) of the RNeasy kit from QIAGEN (Courtaboeuf, France), which includes treatment with RNase-free Dnase (QIAGEN, Courtaboeuf, France), and then subjected to RT-PCR using the cloning primers described above: Oleosin, sense: 5′-CGC GGA TCC ACC ATG GCT ACT GCT ACT GAT CGT-3′, antisense: 5′-CGC GGA TCC TTA TGA TGA TGA CCT CTT AAC ATC-3′; TC-O, sense: 5′-CCC CTA CTT AAC CTC CGT GA-3′, antisense: 5′-GGA GAG GAG CAA CAG AGT GC-3′; O-TC: sense: 5′-CGA CAG GTA CAT GGG ACG AC-3′, antisense: 5′-CCT AGG AGG CAC TGC TGG TA-3′; GFP-TC-O, sense: 5′-ATG CTG AGC AAG GGC-3′, antisense: 5′-CTT GTA CAG CTC GTC-3′. RT-PCR was performed using the Qiagen omniscript Reverse Transcription (Courtaboeuf, France) and Taq platinum (Invitrogen, Cergy Pontoise, France) according to the manufacturer's instruction.

### Cell culture conditions

Caco2/TC7 (human colon adenocarcinoma), COS-7 (immortalized kidney cells of the African green monkey), CHO (Chinese hamster ovary) and HEK (human embryonic kidney) were cultivated in Dulbecco's modified Eagle's medium (DMEM) containing 5% fetal bovine serum (FCS). The Caco-2/TC7 polarized cells are cloned cells established from the human enterocyte-like Caco-2 cell line [31]. NIE 115 mouse neuroblastoma cells chosen here as a neuronal model [32] were cultured in a complete growth medium containing 90% Dulbecco's modified Eagle's medium with 4.5 g/L glucose (supplemented with sodium pyruvate) and 10% fetal bovine serum. The cells were studied in exponential growth and stationary phases. Cell number was measured by the Dye uptake method using Neutral Red cell incorporation. The results were expressed as the percentage of the maximal O.D. value.

### Sodium dodecyl sulfate polyacrylamide gel electrophoresis (SDS-PAGE) and immunoblots of recombinant proteins

were performed using a Mini-protean II electrophoresis unit (Bio-Rad, Hercules, CA, USA). The stacking and the separating gel contained 4% and 12% of acrylamide, respectively. Proteins were separated for 2 hours at 125 V in 25 mM Tris –HCl (pH 8.3), 0.192 M glycine, 0.1% SDS (w/v) and electrotransferred onto nitrocellulose membrane (0.2 µm, Bio-Rad) in 25 mM Tris buffer containing 192 mM glycine and 20% (v/v) methanol (pH 8.3), using a Trans-blot semi-dry transfer cell (Bio-Rad) during 30 minutes at 15 V. The membrane was blocked by incubation in 20 mM Tris-HCl (pH 7.4), 0.15 mM NaCl (TBS), 3% (w/v) BSA for 2 hours at room temperature and was then washed 2 times in 20 mM Tris-HCl buffer containing 0.15 M NaCl (TBS) with 0.05% (v/v) Tween 20. Nitrocellulose was then probed overnight with the anti-oleosin polyclonal rabbit antibody produced and tested by the laboratory (diluted 1∶10 in TBS with 0.1% Tween 20). Anti rabbit HRP-IgG (diluted 1∶5000, Sigma, St Louis, MO, USA). was used for detection with ECL PLUS reagent (Amersham, UK).

### Measurement of binding to radiolabeled cobalamin

Confluent cells were rinsed twice with Dulbecco's modified Eagle medium (DMEM) without the addition of FCS. The cells (10^6^ per assay) were then incubated with 0.48 kBq of [^57^Co]-labeled vitamin B12 (ICN Pharmaceuticals, Orsay, France) for 20 minutes at 4°C. They were rinsed three times with PBS prior to being counted. The resulting radioactivity in the cell pellet was measured in a multi-well gamma counter. The values obtained were used to report the amount of B12 bound to the intact cells. In a parallel series, the cells were lyzed at 4°C in a saline buffer TBS with the presence of protease inhibitors by passing trough a 20-gauge needle fitted to a 1 mL syringe during one minute. Half of the samples were sonicated 3 times for 10 s using a cell disruptor (B-30, Branson Sonic Power Co., USA). After centrifugation (18,000 g, 4°C) for 30 min the cell pellets were incubated with 0.48 kBq of [^57^Co]-labeled vitamin B12 during 20 minutes at 4°C. After three rinses with TBS, partition of radioactivity was determined after centrifugation by measuring [^57^Co]-Cbl found in the pellet vs. that remaining in the supernatant using a gamma-counter (LKB, Wallac, USA). The variation coefficients of the chimer expression (radioactive B12 binding in membrane fraction) within and between the different series of experiments were lower than 20%.

### Intracellular location of the chimers by immuno-histochemistry

Transiently transfected COS-7 cells were used to show TC protein expression and its cellular localization. For immuno-staining, cultured cells were washed once with PBS and fixed with 4% para-formaldehyde and permeabilized with PBS-0.1 Triton X-100 for 30 min. After blocking the nonspecific binding sites with 5% horse serum dissolved in PBS–0.1% Triton X-100 for 30 min at room temperature (RT), the cells were incubated with the primary antibody over night at 4°C. The primary antibodies were, a goat polyclonal antibody anti-TC (1∶50 dilution; Santa Cruz Biotechnology, Santa Cruz, CA, USA), a mouse monoclonal antibody anti-human golgin-97 (1∶50 dilution; Molecular Probes Inc, Eugene, OR, USA) and a rabbit polyclonal antibody anti-calreticulin (1∶100 dilution; Bioreagents, Golden, CO, USA). After washing with PBS, cells were incubated for 2 h at RT with the suitable secondary antibody. These antibodies were, donkey IgG anti-goat fluorescein (FITC) labeled (1∶60 dilution; Jackson ImmunoResearch; Palo Alto, CA, USA), donkey IgG anti-mouse TRITC labeled (1∶60 dilution; Jackson ImmunoResearch; Palo Alto, CA, USA), donkey IgG anti-rabbit FITC labeled (1∶60 dilution; Sigma; St. Louis, MO, USA), and donkey IgG anti-rabbit TRITC labeled (1∶60 dilution; Jackson ImmunoResearch; Palo Alto, CA, USA). After 5-min washing with PBS, the cells were counterstaining with 1 µM Hoechst 33258, washed, and mounted on glass slides using Vectashield (Vector Laboratories; Burlingame, CA, USA). The fluorescence was observed with a Leica DMIRE2 microscope using 5X, 20X, or 40X objectives and the filters A for Hoechst 33258, K3 for FITC. The images were digitized with a Leica DC300F camera (Nussloch, Germany). Negative controls were obtained by replacing the primary antibody by an irrelevant antibody of the same IgG subclass. Fluorescence labeling was also viewed through a multispectral confocal laser-scanning microscope (TCS-SPE, Leica, Heidelberg) using a 60X oil-immersion objective at excitation-emission wavelengths of 405–465 nm (blue channel), 488–522 nm (green channel), and 568–635 nm (red channel). Twenty to forty consecutive optical sections at 0.3-µm intervals were obtained in the z-series. The resulting images were projected on a bidimensional plane and were overlapped on the screen monitor using blue for Hoechst 33258, green for GFP and FITC, and red for TRITC.

The co-localization of TC with endoplasmic reticulum in Caco-2 was further studied in electron microscope immunocytochemistry (transmission electron microscope Siemens 102). Cell pellets were fixed and ultrathin sections were processed as described [33]. The anti-TC was used at a dilution of 1∶25 and the secondary antibody at 1∶50 (Gold conjugate, anti-rabbit IgG, Ted Pella Inc., USA).

For the study of co-localization of megalin with reticulum in Caco-2, we used a Leica TCS SP2-AOBS confocal microscope (Leica Microsystems, Germany) equipped with an acousto-optical beamsplitter, an argon laser (488 nm), an Helium Neon laser (543 nm) were respectively used with an HCX APO L x40/0.8. The bandwidths of the detected fluorescence wavelengths have been optimized for each channel to the maximum emission (502–530 for Alexa488 and 550–570 nm for Alexa555 respectively with 567 V and 463 V PMT Gain). All regular acquisitions were collected at 400 Hz in sequential sequences to avoid potential cross talking. Fluorescence emissions were recorded within 1 Airy disk confocal pinhole opening and images at a 0. 238 µm (x, y) pixel size were obtained for each case in 1024×1024 matrices (fields of 158,94 µm2). Mouse Anti-human Megalin is from Array Genetics (USA). Rabbit anti-human calnexin (Sigma-Aldrich, France) was used as a specific marker of reticulum in caco-2 cells.

### Intracellular conversion of exogenous [57Co]-labelled B12 (cyano-cobalamin, CN-Cbl)

Cells were plated onto 100 mm dishes.^57^Co-labeled vitamin B12 (11 MBq/nmol) was prepared and incorporated into culture medium (0.37 pmol, 3.88 kBq/dish) and added in dishes containing 80–90% confluent cells, as described previously [8]. The final concentration of B12 in medium was estimated to 100 pmol. The loading was repeated every 24 hrs and cells were harvested for HPLC analysis of CN-cobalamin, ado-cobalamin and methyl-cobalamin after 72 hrs. The uptake of the radioactive B12 was normalized to the protein content of each sample collected from independent 100 mm dishes. The various forms of vitamin B12 in cytosolic and mitochondria enriched fractions were analysed by HPLC as described [8].

### S-Adenosylmethionine (SAM) and S-Adenosylhomocysteine (SAH)

the method was adapted from Miller et al. [34] as described [35] and is based on a reverse phase high performance liquid chromatography technique using a linear acetonitrile gradient. Briefly, cell pellet was first washed with 1X PBS (Sigma) and then lysed with the following lysis buffer: 0.05 M NaH2PO4 (pH 8), 0.15 M NaCl, 0.1 M Imidazole, 0.5% Chaps and Complete™ (Roche) Protease Inhibitors. The extracted proteins were precipitated with 3% HClO4 and centrifuged at 12,000 g at 4°C for 10 minutes. The supernatant was passed through a 0.45 µm filter before injection into the column (Lichrospher®, C18, OD 2.5 µm, 250×4 mm I.D). The mobile phase was maintained at 30°C and applied at a flow rate of 0.75 mL/min at 105 bars. It consisted of 50 mM sodium phosphate (pH 3.2), 10 mM heptan sulfonic acid and acetonitrile (10 to 20% from 0 to 20 minutes). The amount of SAM and SAH were quantified by absorbance at 254 nm.

### Measurement of methionine synthase activity

Cells were seeded at a density of 6×10^3^ cells/cm^2^ onto 100 mm Petri dishes and cultured until confluence. Determination of methionine synthase activity was performed by a modification of the radioisotope assay described by Weissbach [36] and modified by us [37]. In brief, cell lysates (1 mg) were homogenized at 4°C in 0.5 ml of 0.1 M potassium phosphate buffer (pH 7.2) in the presence of protease inhibitors. After centrifugation (10,000 g, 4°C) for 3 min to remove cell debris, the supernatant was used as crude extract. For measurement of apoenzyme activity, reaction mixture contained 0.25 mM DL-homocysteine, 29 mM dithiotreitol (used as reducing agent), 7 mM β-mercaptoethanol, 0.25 mM S-adenosyl-methionine, 37 kBq (22 µM) [methyl-^14^C]MeH_4_F, crude extract and 50 µM potassium phosphate buffer in a total volume of 800 µl. The enzyme reaction was carried out under a N_2_ atmosphere at 37°C for 1 h in the dark and then stopped by heating at 95°C for 5 min. The assay measures the amount of radioactive methionine formed from 5-[^14^C]methyl-THF and Hcy. The mixture was passed through AG-1X8 (Cl-) columns (Bio-Rad, Marnes-la-Coquette, France) and ^14^C radioactivity in the methionine-containing fraction was measured. The radiolabeled methionine was measured in a Packard liquid scintillation counter. Enzyme activity was expressed as nmoles of methionine produced per hour per mg of proteins.

### Statistics

All data were normally distributed and therefore expressed as means±standard deviation (S.D.). The significance between the various transfected cell lines and the effects of culture conditions was determined by analysis of variance (ANOVA). In all analyses, the null hypothesis was rejected at the 0.05 level.
